# Fingerroot Extract Oral Spray for Anticariogenic Purpose: Integration of Ultrasound‐Assisted Extraction and Solvent System Optimization

**DOI:** 10.1155/adpp/5407088

**Published:** 2026-06-28

**Authors:** Chaowalit Monton, Thaniya Wunnakup, Jirapornchai Suksaeree, Laksana Charoenchai

**Affiliations:** ^1^ Drug and Herbal Product Research and Development Center, College of Pharmacy, Rangsit University, Pathum Thani, 12000, Thailand, rsu.ac.th; ^2^ Department of Pharmacognosy, College of Pharmacy, Rangsit University, Pathum Thani, 12000, Thailand, rsu.ac.th; ^3^ Medicinal Cannabis Research Institute, College of Pharmacy, Rangsit University, Pathum Thani, 12000, Thailand, rsu.ac.th; ^4^ Department of Pharmaceutical Chemistry, College of Pharmacy, Rangsit University, Pathum Thani, 12000, Thailand, rsu.ac.th

**Keywords:** *Boesenbergia rotunda*, Box–Behnken design, dental caries, I-optimal design, *Streptococcus mutans*

## Abstract

Fingerroot (*Boesenbergia rotunda* (L.) Mansf.), a member of the Zingiberaceae family, exhibits anticariogenic activity against *Streptococcus mutans*. This study aimed to optimize ultrasound‐assisted extraction and solvent system selection for oral spray development. Extraction conditions were optimized using a Box–Behnken design, evaluating ultrasonication time, solvent‐to‐solid ratio, and ethanol concentration. The optimal conditions—45‐min ultrasonication, 30:1 solvent‐to‐solid ratio, and 85% ethanol—yielded the highest contents of pinocembrin, pinostrobin, and panduratin A. The optimized extract was then subjected to solvent system optimization using an I‐optimal design to identify combinations of water, polyethylene glycol 400, and 95% ethanol for effective flavonoid recovery and solubilization. Three solvent systems (12:13:75, 10:30:60, and 6:60:34) were selected for oral spray formulation. Each formulation contained 0.03% sucralose, 0.3% menthol, 0.02% sodium methylparaben, and 0.18% sodium propylparaben. The resulting sprays demonstrated anticariogenic activity against *S. mutans*. Stability studies showed a decline in pH and flavonoid content over time, whereas viscosity and antimicrobial activity remained stable, as evidenced by inhibition zones. In summary, this study successfully combined extraction and solubilization optimization strategies to develop a fingerroot oral spray with potential anticariogenic properties.

## 1. Introduction

Dental caries, often known as cavities or tooth decay, is the most common noncommunicable disease in the world. Severe dental caries can have a major negative influence on general health by causing infections and pain that may require tooth extraction [1]. The primary cause of it is an accumulation of the cariogenic bacterium *Streptococcus mutans* in the oral cavity [2]. Tooth enamel becomes demineralized as a result of this bacterium’s ability to ferment dietary carbohydrates and turn them into acid. While preventive measures, such as fluoride treatments, brushing with antimicrobial toothpaste, and regular dental visits, have reduced the prevalence of dental caries, the disease remains a significant public health concern [3]. The increasing resistance of *S. mutans* to synthetic antimicrobial agents has also raised concerns [4], highlighting the need for alternative, natural, and sustainable approaches to caries prevention.

Given these challenges, there has been a growing interest in the development of natural oral care products derived from plants that possess antimicrobial and anticariogenic properties. Among the numerous plants studied for their potential oral health benefits, *Boesenbergia rotunda* (L.) Mansf., commonly known as fingerroot, has attracted attention due to its diverse range of bioactive compounds and promising pharmacological activities. This herb, belonging to the Zingiberaceae family, has been used for centuries in Southeast Asia for its medicinal properties, particularly for its antimicrobial, anti‐inflammatory, and antioxidant effects [5–7]. Studies have indicated that fingerroot exhibits significant activity against various microbial pathogens, including *S. mutans*, the primary causative agent of dental caries [6]. Fingerroot contains a variety of bioactive flavonoids, such as pinocembrin (PC), pinostrobin (PS), and panduratin A (PA), which have been shown to inhibit the growth of oral pathogens and reduce the formation of dental biofilms [8–10].

Despite the promising antimicrobial properties of fingerroot, the extraction of bioactive compounds from this plant remains a critical challenge. The effectiveness of a fingerroot‐based oral care product largely depends on the efficiency of the extraction process, as it determines the yield and quality of bioactive compounds. Traditional extraction methods, such as maceration or reflux extraction, often require long extraction times and large quantities of solvents, which can lead to the degradation of sensitive compounds and lower extraction yields [11, 12]. In contrast, ultrasound‐assisted extraction (UAE) has emerged as a promising technique to improve the extraction efficiency of bioactive compounds from plant materials. The UAE uses high‐frequency sound waves to induce cavitation, which enhances the penetration of the solvent into the plant matrix and facilitates the release of bioactive compounds. This method has been shown to reduce extraction time, minimize solvent use, and preserve the integrity of sensitive compounds, making it an attractive option for the extraction of bioactive compounds from fingerroot [12].

An oral spray formulation represents a promising delivery system for bioactive extracts, particularly in the context of oral care applications. Oral sprays offer advantages in terms of convenience, ease of administration, and the potential for rapid delivery of active compounds to the oral cavity [13, 14]. This mode of delivery enables targeted application to the teeth and gingival tissues, where *S*. *mutans* is primarily located [2]. Given the documented antimicrobial properties of fingerroot, incorporating its bioactive extract into an oral spray formulation may facilitate direct delivery of active constituents to the site of microbial colonization. Such a formulation could serve as an effective, natural alternative to conventional synthetic oral care products. Although various herbal extracts and antimicrobial agents have been incorporated into oral care products, most previous studies have primarily focused on antimicrobial activity or extract characterization rather than formulation development. In addition, studies specifically investigating oral spray formulations for anticariogenic applications remain limited, particularly those integrating extraction optimization, solvent system optimization, and formulation design within a single study framework. Moreover, formulation‐related considerations, such as flavonoid solubilization, physicochemical stability, and delivery suitability for oral spray systems, are often insufficiently addressed. Therefore, the present study aimed to bridge these gaps by integrating extraction optimization and solvent system development into the preparation of a fingerroot extract oral spray with potential anticariogenic activity against *S. mutans*.

The ultimate goal of this study is to integrate extraction optimization, solvent system optimization, and formulation development to produce a natural oral spray containing fingerroot extract with potential anticariogenic activity against *S. mutans*. This study aims to optimize both the UAE process and the solvent system used to solubilize the extract, to enhance the extraction yield of key bioactive flavonoids and maximize their solubility in the final formulation. By systematically optimizing these two critical issues, the study seeks to develop a formulation that is effective in preventing dental caries for use in daily oral care. The integration of the UAE and solvent system optimization offers a promising approach to improving the extraction and formulation of fingerroot‐based products, potentially leading to a natural alternative for the prevention and management of dental caries.

## 2. Materials and Methods

### 2.1. Materials

PC and PS standards were purchased from Chengdu Biopurify Phytochemicals Ltd., Sichuan, China. PA standard (PhytoLab) was purchased from Chemical Express Co., Ltd., Samut Prakan, Thailand. Glycerin, propylene glycol (PG), polyethylene glycol (PEG) 400, sucralose, and 95% ethanol were purchased from Krungthepchemi Co., Ltd., Thailand. Menthol was purchased from P.C. Drug Center, Bangkok, Thailand. Sodium methylparaben and sodium propylparaben were purchased from Namsiang Co., Ltd., Bangkok, Thailand. Ultrapure water was produced from a Direct‐Q 3UV water purifier (Merck KGaA, Darmstadt, Germany). Media and chemicals related to antimicrobial testing were purchased from HiMedia Laboratories LLC, PA, USA. All AR‐grade and HPLC‐grade solvents (Fisher Chemical) were purchased from Apex Chemicals Co., Ltd., Bangkok, Thailand.

### 2.2. Fingerroot Sample Collection and Preparation

The fingerroot was collected from Ban Apon, Buached District, Surin Province, Thailand (14°29′47″N, 103°55′35″E; 190 m elevation), with Global Positioning System (GPS) coordinates recorded using an iPhone 13 Pro, during the harvesting period on May 25–26, 2024. The source of fingerroot was selected based on its previously demonstrated high flavonoid content [5]. The plant sample was identified by Asst. Prof. Dr. Orawan Theanphong, Department of Pharmacognosy, College of Pharmacy, Rangsit University. A voucher specimen (No. TMRC 067) was deposited at the Drug and Herbal Product Research and Development Center, College of Pharmacy, Rangsit University. The fingerroot was separated into root and rhizome portions, which were then washed with tap water, sliced to a thickness of 2–3 mm, air‐dried for 2 h, and subsequently dried in a circulating hot‐air oven at 60°C for 6 h (roots) and 8 h (rhizomes). The dried materials were combined, pulverized, and then passed through a 40‐mesh sieve.

### 2.3. Optimization of UAE of Fingerroot Powder

Fingerroot powder was extracted using the UAE technique with an ultrasonic bath operating at a frequency of 40 kHz and an ultrasonic power of 120 W (VGT‐1730QTD, GT Sonic, Guangdong, China). Fingerroot powder (1 g) and a specific concentration of ethanol at a defined solvent‐to‐solid ratio were added to a 250‐mL Erlenmeyer flask and covered with aluminum foil. The mixture was then ultrasonicated for a specified time and temperature. The steps for screening factor levels based on the one‐factor‐at‐a‐time approach are shown in Table 1. The resulting solution was filtered using a nylon syringe filter with a 0.45‐μm pore size and analyzed for the contents of PC, PS, and PA under validated HPLC conditions as described in a previous study [5].

**TABLE 1:** Steps for screening factor levels for the UAE of fingerroot.

Step	Ultrasonication time (min)	Temperature (°C)	Solid‐to‐solvent ratio (g/mL)	Ethanol concentration (% v/v)
1	15, 30, 45, 60, and 120	40	1:20	95
2	45	40, 50, 60, 70, and 80	1:20	95
3	45	50	1:10, 1:20, 1:30, 1:40, and 1:50	95
4	45	50	1:20	50, 60, 70, 80, 95, and 100

The factor levels obtained from the screening step were incorporated into the Box–Behnken design. Three factors—ultrasonication time, solvent‐to‐solid ratio, and ethanol concentration—were selected and designed using Design‐Expert Version 11 (Stat‐Ease Inc., MN, USA). Each factor was varied at three levels, as shown in Table 2. Fingerroot powder was extracted using the aforementioned procedures, followed by HPLC analysis of PC, PS, and PA contents. The percentages of PC, PS, and PA were calculated based on the dry weight of fingerroot powder. Response surface plots were generated, and analysis of variance (ANOVA) was conducted. The optimization criteria were set to simultaneously maximize the contents of PC, PS, and PA. The optimal conditions were used to extract fingerroot powder in three independent runs to verify the predictive accuracy of the Design‐Expert model. Predictive accuracy was evaluated based on percentage errors, calculated by comparing the experimental values with the predicted values.

**TABLE 2:** Factor levels of the investigated factors—ultrasonication time, solvent‐to‐solid ratio, and ethanol concentration—used in the Box–Behnken design.

Factors	Levels		
	−1	0	+1
Ultrasonication time (min)	45	82.5	120
Solvent‐to‐solid ratio (mL/g)	10:1	20:1	30:1
Ethanol concentration (% v/v)	75	80	85

### 2.4. Scanning Electron Microscopy (SEM) Analysis of Fingerroot Powder

Fingerroot samples before and after treatment with the optimal UAE condition were gold‐coated and subsequently examined using SEM (Hitachi S‐3400N Type II, Hitachi, Tokyo, Japan) at magnifications of 100× and 500×. The microstructures of the fingerroot samples were compared.

### 2.5. Extraction of Fingerroot for Optimization of Solvent System and Oral Spray Preparation

Fingerroot extract was prepared by adding 500 mL of 85% ethanol to a 2‐L beaker containing 250 g of fingerroot powder, followed by ultrasonication for 120 min without temperature control. This solvent‐to‐solid ratio and extended extraction time were chosen to achieve flavonoid yields comparable to the previously optimized UAE while minimizing solvent use and processing time for solvent removal. This process was performed in four sets, using a total of 1 kg of fingerroot powder. The resulting solution was filtered using vacuum filtration, followed by ethanol removal through vacuum rotary evaporation at 45°C. The extract was then freeze‐dried overnight using a freeze dryer (LFD‐12D, Laboao, Henan, China) to remove residual water.

### 2.6. Antimicrobial Activity of the Optimal Fingerroot Extract Against *S. mutans*

The minimum inhibitory concentration (MIC) and minimum bactericidal concentration (MBC) of fingerroot extracts against *S. mutans* ATCC 25175 were assessed to identify the appropriate concentration of fingerroot extract for incorporation in an oral spray formulation. Initially, *S. mutans* was cultured overnight in brain heart infusion (BHI) broth under anaerobic conditions at 37°C. The culture was subsequently diluted in BHI broth to conform to the 0.5 McFarland standard and further diluted 100‐fold to reach a concentration of 1 × 10^6^ CFU/mL. Fingerroot extracts were prepared at a concentration of 200 mg/mL in 85% ethanol and then diluted in BHI broth to create final concentrations ranging from 2 to 1024 μg/mL. A 100‐μL aliquot of the fingerroot extracts was combined with 100 μL of the bacterial suspension in each well of a sterile 96‐well plate (*n* = 3). The plates were incubated anaerobically at 37°C for 24 h. Following this incubation period, 10 μL of 1 mg/mL resazurin was added to each well, and the plates were incubated for an additional 2–4 h at 37°C under aerobic conditions. The MIC was determined as the lowest concentration of fingerroot extracts at which the solution remained blue, indicating inhibition of bacterial growth; a color change from blue to pink indicates bacterial growth and reduction of resazurin to resorufin [15]. Subsequently, 10 μL of these blue solutions was transferred onto BHI agar (*n* = 3) and incubated anaerobically at 37°C for 24 h. The MBC was defined as the lowest concentration of fingerroot extracts that yielded no bacterial growth following incubation.

### 2.7. Solvent Selection for Solubilization of Fingerroot Extract

Fingerroot extract was solubilized in various solvents to evaluate its potential use as a solvent for oral spray formulations. The extract was dissolved in water, glycerin, PG, PEG 400, or 95% ethanol to a final concentration of 1% w/w in 2‐mL microfuge tubes (*n* = 3). The samples were mixed using a vortex mixer (Vortex Genie 2, Scientific Industries, Inc., NY, USA) for 1 min, ultrasonicated for 10 min, and then centrifuged at 6000 rpm for 5 min (IKA mini G, IKA Works [Thailand] Co. Ltd., Bangkok, Thailand). The physical appearance, including clarity, turbidity, sedimentation, and phase separation, was observed. The supernatant from the center of each tube was sampled, diluted to 200 mg/5 mL using methanol, filtered, and analyzed for PC, PS, and PA contents using HPLC. The percent recoveries of all flavonoids were calculated relative to the 85% ethanol solvent, which served as the initial extraction solvent for fingerroot.

### 2.8. Optimization of the Solvent System for the Solubilization of Fingerroot Extract

The mass fractions of water, PEG 400, and 95% ethanol were determined using an I‐optimal mixture design in Design‐Expert, with the following constraints applied:

(1) 00.3≤Water≤,

(2) 04001.0≤PEG ≤,

(3) 0951.0≤% ethanol≤,

(4) Water+PEG 400951.0+% ethanol=.

Fingerroot extract was solubilized using the aforementioned procedures, followed by HPLC analysis of PC, PS, and PA contents. The solubility characteristics and percentage recoveries of PC, PS, and PA were reported. Contour plots were generated, and ANOVA was performed. The optimization criteria to produce design space were set to a clear solution and recoveries of PC, PS, and PA of at least 90%. A design space was established, from which three optimal solvent systems were selected. Fingerroot solutions were then prepared in three independent runs for each optimal solvent system to verify the predictive accuracy of the Design‐Expert model. Predictive accuracy was assessed based on percentage errors, calculated by comparing the experimental values with the predicted values.

### 2.9. Formulation and Evaluation of an Oral Spray Containing Fingerroot Extract

Oral spray formulations were prepared using different optimal solvent systems. Each formulation, expressed as a mass ratio, consisted of 1% fingerroot extract, 0.03% sucralose as a sweetening agent, 0.3% menthol as a flavoring agent, 0.02% sodium methylparaben and 0.18% sodium propylparaben as preservatives, and 98.47% optimal solvent as the vehicle. The preparation involved solubilizing the fingerroot extract, menthol, and PEG 400 in 95% ethanol. A separate aqueous phase was prepared by dissolving sucralose, sodium methylparaben, and sodium propylparaben in water. The aqueous phase was then gradually added to the ethanol phase with continuous mixing, followed by ultrasonication for 10 min to ensure complete solubilization. The excipients were selected based on their common use in oral pharmaceutical formulations to provide sweetness, flavor, preservation, and solubilization functions. However, potential interactions between excipients and active phytochemicals, as well as sensory acceptability, were not investigated in the present study.

The three optimal oral spray formulations were evaluated for pH, viscosity, key flavonoid contents, and clear zone against *S. mutans*. Briefly, the pH of each formulation was measured in triplicate using a pH meter (SevenCompact S220, Mettler Toledo, Greifensee, Switzerland). Viscosity was determined using a viscometer (IKA ROTAVISC hi‐vi I, IKA Works [Thailand] Co. Ltd., Bangkok, Thailand) equipped with spindle no. SP‐8 at 100 rpm, with measurements taken three times at 10‐s intervals. The contents of key flavonoids were analyzed by diluting 200 mg of each formulation with methanol in a 5‐mL volumetric flask (*n* = 3), followed by HPLC analysis. Finally, the clear zone against *S. mutans* was assessed using the agar well diffusion method, with 40 μL of the oral spray added to each well (*n* = 3), following a procedure similar to that described previously.

### 2.10. Stability Assessment of the Optimized Fingerroot Extract Oral Spray

The three optimal oral spray formulations were stored in amber bottles with caps and subjected to stability testing in a climate chamber (Memmert GmbH + Co. KG, Schwabach, Germany) under two conditions: 30°C/75% RH and 40°C/75% RH for 3 months. The pH, viscosity, key flavonoid contents, and clear zone against *S. mutans* were evaluated and compared to their respective values at the initial time point.

### 2.11. Statistical Analysis

The data were analyzed using an independent‐sample *t*‐test to determine differences between two groups or ANOVA followed by a least significant difference (LSD) post hoc analysis to determine the differences among multiple groups. This analysis was conducted using IBM SPSS Statistics Version 22 (IBM Corporation, NY, USA). A *p* value of less than 0.05 was regarded as statistically significant at the 95% confidence interval.

## 3. Results and Discussion

### 3.1. Optimal UAE Condition

Flavonoids, particularly PC and PA, have been reported to inhibit the growth of *S. mutans* [9, 10]. Previously, the UAE was employed to extract fingerroot under varying temperatures and durations; however, the focus was primarily on antioxidant activity [16]. In contrast, this study emphasizes individual flavonoids—PC, PS, and PA—and their anticariogenic activity against *S. mutans* to enhance the value of this herbal plant.

Ultrasonication time is a critical variable under investigation. In general, it influences extraction efficiency by enhancing mass transfer and disrupting plant cell walls, thereby facilitating the release of bioactive compounds [17]. Extraction temperature is a key factor influencing extraction efficiency. Typically, elevated temperatures enhance extraction yield by increasing the diffusion rate and solubility of phytochemicals in the solvent, while also promoting better solvent penetration into plant matrices by lowering the solvent’s viscosity and surface tension at the interface of the solvent, solutes, and plant material [18]. However, excessively high temperatures may lead to the degradation of bioactive compounds in the plant material [19]. The solvent‐to‐solid ratio is an important variable influencing extraction efficiency. A higher ratio increases the concentration gradient between the solvent and the solid, thereby enhancing the diffusion rate of compounds. However, it may also prolong the time required to reach equilibrium. This variable significantly affects the extraction yield, typically resulting in a rapid increase followed by a plateau as maximum yield is approached [20]. Ethanol concentration plays a critical role in the solubilization of bioactive compounds [21]. Solvent type and concentration directly influence extraction efficiency, primarily depending on the polarity of the target compounds. Additionally, factors, such as environmental safety, human toxicity, and cost‐effectiveness, must be considered when selecting an appropriate solvent system [22].

**FIGURE 1:**
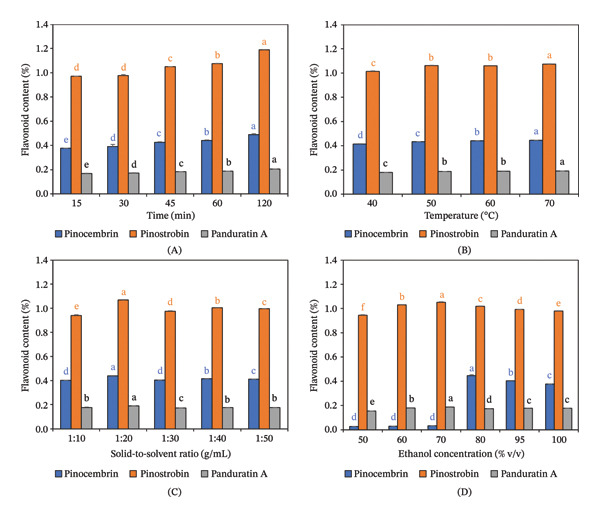
Effect of (A) ultrasonication time, (B) temperature, (C) solid‐to‐solvent ratio, and (D) ethanol concentration on the contents of PC, PS, and PA in fingerroot powder extracted using the UAE method. Different letters on the bar graph indicate statistically significant differences (*p* < 0.05) among extraction conditions for the same compound.

Firstly, key factors influencing the extraction efficiency of UAE—including ultrasonication time, temperature, solid‐to‐solvent ratio, and ethanol concentration—were screened using a one‐factor‐at‐a‐time approach. The results are presented in Figure 1. An increase in ultrasonication time from 15 to 120 min (Figure 1A) or temperature from 40°C to 70°C (Figure 1B) significantly enhanced the levels of PC, PS, and PA. Notably, the highest levels of these flavonoids were observed at a solid‐to‐solvent ratio of 1:20 when the ratio was varied from 1:10 to 1:50 (Figure 1C). Regarding ethanol concentration, it has been reported that ethanol is more effective than aqueous solvents for flavonoid extraction [5]; therefore, hydroethanolic solvents containing at least 50% ethanol were used. In this study, the maximum PC content was achieved at 80% ethanol, while PS and PA were maximized at 70% ethanol (Figure 1D).

Among these variables, all except temperature were selected for subsequent experiments. Temperature was excluded due to its relatively minor impact on extraction efficiency compared to the other factors. Additionally, a limitation of this study is that the ultrasonic bath used was unable to maintain a constant temperature throughout the extraction process, as it could only control the initial temperature. This is attributed to the heat generated by the cavitation mechanism during UAE [12].

Ultrasonication times ranging from 45 to 120 min, solvent‐to‐solid ratios from 10:1 to 30:1, and ethanol concentrations between 75% v/v and 85% v/v were incorporated into the Box–Behnken design. The factors and corresponding responses for the UAE of fingerroot are presented in Table 3.

**TABLE 3:** Factors and responses of the Box–Behnken design for the UAE of fingerroot.

Std	Run	Factors			Responses (*n* = 3)		
		Time (min)	Solvent‐to‐solid ratio (mL/g)	Ethanol concentration (%)	PC content (%)	PS content (%)	PA content (%)
13	1	82.5	20:1	80	0.73 ± 0.01	2.05 ± 0.02	0.99 ± 0.02
16	2	82.5	20:1	80	0.72 ± 0.02	2.02 ± 0.06	0.97 ± 0.03
4	3	120	30:1	80	0.72 ± 0.03	2.03 ± 0.10	0.97 ± 0.05
15	4	82.5	20:1	80	0.72 ± 0.04	2.04 ± 0.13	0.97 ± 0.05
10	5	82.5	30:1	75	0.74 ± 0.06	2.14 ± 0.17	1.05 ± 0.08
8	6	120	20:1	85	0.71 ± 0.04	2.17 ± 0.12	1.04 ± 0.04
5	7	45	20:1	75	0.73 ± 0.06	2.18 ± 0.14	1.03 ± 0.07
7	8	45	20:1	85	0.76 ± 0.04	2.23 ± 0.16	1.07 ± 0.08
3	9	45	30:1	80	0.79 ± 0.05	2.26 ± 0.13	1.09 ± 0.07
1	10	45	10:1	80	0.71 ± 0.02	1.91 ± 0.09	1.03 ± 0.05
11	11	82.5	10:1	85	0.77 ± 0.04	2.02 ± 0.10	1.08 ± 0.06
2	12	120	10:1	80	0.78 ± 0.03	2.06 ± 0.10	1.10 ± 0.05
9	13	82.5	10:1	75	0.74 ± 0.03	1.99 ± 0.09	1.04 ± 0.05
17	14	82.5	20:1	80	0.75 ± 0.06	2.15 ± 0.17	1.02 ± 0.07
14	15	82.5	20:1	80	0.75 ± 0.04	2.13 ± 0.12	1.03 ± 0.05
12	16	82.5	30:1	85	0.72 ± 0.06	2.17 ± 0.18	1.05 ± 0.08
6	17	120	20:1	75	0.74 ± 0.05	2.17 ± 0.13	1.03 ± 0.08

**FIGURE 2:**
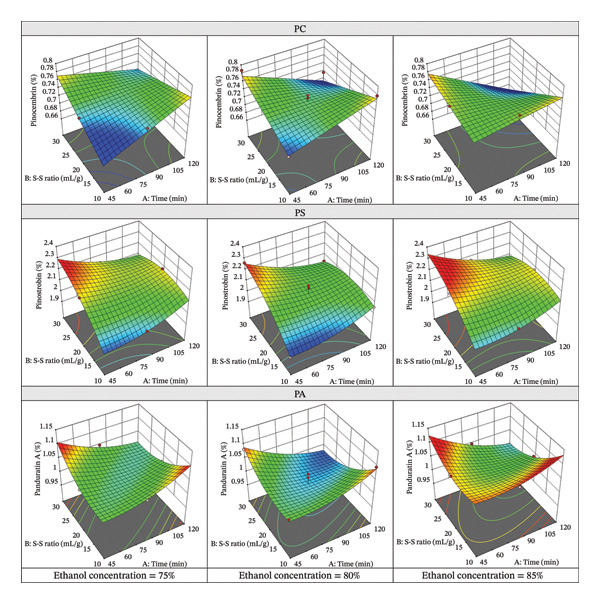
Response surface plots of PC (top), PS (middle), and PA (bottom) illustrating the effects of ultrasonication time and solvent‐to‐solid ratio at varying ethanol concentrations: 75% (left), 80% (middle), and 85% (right).

Response surface plots of PC, PS, and PA are shown in Figure 2, and the corresponding ANOVA data are presented in Table 4. The PC content was fitted to a two‐factor interaction model, in which increasing the ultrasonication time at a low solvent‐to‐solid ratio resulted in higher PC content, whereas increasing the ultrasonication time at a high solvent‐to‐solid ratio led to a decrease in PC content. Similarly, increasing the solvent‐to‐solid ratio at shorter ultrasonication times enhanced PC content, while higher ratios at longer ultrasonication times reduced it. The only significant term influencing PC content was the interaction between time and solvent‐to‐solid ratio (*p* = 0.0013).

**TABLE 4:** ANOVA results for the two‐factor interaction model of PC content and the quadratic models of PS and PA contents.

Source	Sum of squares	df	Mean square	*F*‐value	*p* value
*PC content*					
Model	0.0067	6	0.0011	4.7100	0.0157^∗^
A‐time	0.0003	1	0.0003	1.1800	0.3035
B‐S‐S ratio	0.0002	1	0.0002	0.8375	0.3816
C‐EtOH conc.	0.0000	1	0.0000	0.0548	0.8197
AB	0.0046	1	0.0046	19.3700	0.0013^∗^
AC	0.0009	1	0.0009	3.7300	0.0824
BC	0.0007	1	0.0007	3.1100	0.1082
Residual	0.0024	10	0.0002		
Lack of fit	0.0013	6	0.0002	0.8117	0.6106
Pure error	0.0011	4	0.0003		
Cor total	0.0090	16			

*PS content*					
Model	0.1284	9	0.0143	7.2500	0.0080^∗^
A‐time	0.0024	1	0.0024	1.2200	0.3055
B‐S‐S ratio	0.0491	1	0.0491	24.9600	0.0016^∗^
C‐EtOH conc.	0.0015	1	0.0015	0.7559	0.4134
AB	0.0360	1	0.0360	18.2700	0.0037^∗^
AC	0.0006	1	0.0006	0.3056	0.5976
BC	0.0000	1	0.0000	0.0126	0.9137
A^2^	0.0087	1	0.0087	4.4200	0.0736
B^2^	0.0164	1	0.0164	8.3500	0.0233^∗^
C^2^	0.0153	1	0.0153	7.7700	0.0270^∗^
Residual	0.0138	7	0.0020		
Lack of fit	0.0003	3	0.0001	0.0267	0.9932
Pure error	0.0135	4	0.0034		
Cor total	0.1422	16			

*PA content*					
Model	0.0210	9	0.0023	4.6700	0.0273^∗^
A‐time	0.0010	1	0.0010	1.9300	0.2076
B‐S‐S ratio	0.0010	1	0.0010	2.0500	0.1951
C‐EtOH conc.	0.0011	1	0.0011	2.2700	0.1760
AB	0.0076	1	0.0076	15.1900	0.0059^∗^
AC	0.0002	1	0.0002	0.4414	0.5277
BC	0.0004	1	0.0004	0.8562	0.3856
A^2^	0.0016	1	0.0016	3.2500	0.1144
B^2^	0.0038	1	0.0038	7.6800	0.0276^∗^
C^2^	0.0032	1	0.0032	6.3800	0.0394^∗^
Residual	0.0035	7	0.0005		
Lack of fit	0.0004	3	0.0001	0.1629	0.9161
Pure error	0.0031	4	0.0008		
Cor total	0.0245	16			

*Note:* An asterisk (^∗^) denotes a significant value.

The PS content was fitted to a quadratic model. Increasing ultrasonication time led to higher PS content at a low solvent‐to‐solid ratio but resulted in a decrease at a high solvent‐to‐solid ratio. An increase in the solvent‐to‐solid ratio at shorter ultrasonication times appeared to have a more pronounced effect on enhancing PS content compared to longer ultrasonication times. Four terms significantly influenced PS content: solvent‐to‐solid ratio (*p* = 0.0016), the interaction between ultrasonication time and solvent‐to‐solid ratio (*p* = 0.0037), the quadratic term of solvent‐to‐solid ratio (*p* = 0.0233), and the quadratic term of ethanol concentration (*p* = 0.0270).

The PA content was fitted to a quadratic model. Higher PA content was observed with either a long ultrasonication time combined with a low solvent‐to‐solid ratio or a short ultrasonication time combined with a high solvent‐to‐solid ratio. Three terms significantly influenced PA content: the interaction between ultrasonication time and solvent‐to‐solid ratio (*p* = 0.0059), the quadratic term of solvent‐to‐solid ratio (*p* = 0.0276), and the quadratic term of ethanol concentration (*p* = 0.0394).

The optimal condition that simultaneously maximized the contents of PC, PS, and PA was an ultrasonication time of 45 min, a solvent‐to‐solid ratio of 30:1, and an ethanol concentration of 85% v/v, yielding a desirability value of 0.949. Verification experiments demonstrated that the PC, PS, and PA contents obtained from three independent extraction batches closely matched the predicted values, with percentage errors below 10% (Table 5), indicating the high predictive accuracy of the model.

**TABLE 5:** Verification data of UAE of fingerroot, presented as predicted values, experimental values, and the corresponding percentage errors.

Responses	Predicted values	Batches	Experimental values (*n* = 3)	Error (%)^∗^
PC content (%)	0.78	1	0.73 ± 0.01	−6.85
		2	0.72 ± 0.01	−8.33
		3	0.74 ± 0.01	−5.41

PS content (%)	2.34	1	2.14 ± 0.03	−9.35
		2	2.16 ± 0.03	−8.33
		3	2.17 ± 0.02	−7.83

PA content (%)	1.13	1	1.05 ± 0.02	−7.62
		2	1.05 ± 0.02	−7.62
		3	1.07 ± 0.01	−5.61

^∗^Error = (experimental value − predicted value) × 100/experimental value.

The authors acknowledged a limitation of this study in that the ultrasonic bath used was unable to maintain a constant temperature throughout the extraction process. As a result, temperature served as a confounding factor influencing the extraction of fingerroot. However, the authors monitored temperature fluctuations during the experiments: The initial temperature was approximately 30°C, increasing to 50°C–51°C, 55°C–56°C, and 58°C–60°C with ultrasonication times of 45, 82.5, and 120 min, respectively. Nevertheless, this confounding factor appeared to have minimal impact on extraction efficiency, as evidenced by the trends observed in Figure 1B and the low percentage error in predictive accuracy.

**FIGURE 3:**
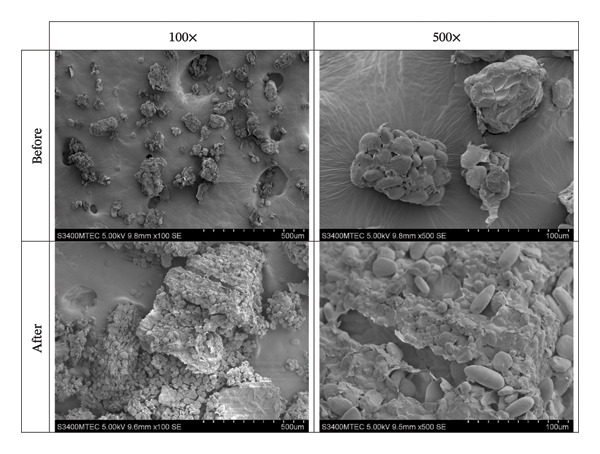
SEM photomicrographs of fingerroot powder before (top) and after (bottom) undergoing UAE treatment, captured at magnifications of 100× (left) and 500× (right).

Photomicrographs of fingerroot samples before and after treatment under the optimal UAE conditions were examined using SEM. SEM images of untreated samples at 100x and 500x magnification revealed numerous parenchyma cells containing densely packed starch grains. In contrast, samples subjected to UAE treatment exhibited a looser parenchyma structure, with dispersed individual starch grains observed (Figure 3). These morphological changes indicate that the cavitation mechanism induced by UAE likely promoted several processes—such as fragmentation, erosion, capillarity, detexturation, and sonoporation—that collectively enhanced extraction efficiency [12].

Fingerroot extract used for subsequent processing was prepared under modified extraction conditions consisting of an ultrasonication time of 120 min, a solvent‐to‐solid ratio of 2:1, and an ethanol concentration of 85% v/v, resulting in an extraction yield of 5.67%. Although these conditions differed from the optimized UAE parameters identified by the Box–Behnken design, the modification was intended to facilitate practical large‐scale extraction by reducing solvent consumption, simplifying handling, and shortening the solvent removal process. The longer ultrasonication time was applied to compensate for the lower solvent volume and to maintain extraction efficiency. Nevertheless, quantitative comparison between the optimized laboratory‐scale extraction condition and the modified large‐scale extraction process was not systematically investigated and should be evaluated in future studies.

### 3.2. Anticariogenic Activity Against *S. mutans* of the Fingerroot Extract

Fingerroot extract exhibited MIC and MBC values against *S. mutans* of 16 and 256 μg/mL, respectively. The MBC/MIC ratio exceeded 4‐6, indicating that the extract possessed bacteriostatic rather than bactericidal activity [23]. The bacteriostatic nature of the fingerroot extract suggests that its anticariogenic effect against *S. mutans* is achieved by inhibiting bacterial growth rather than directly eliminating the microorganisms. For long‐term use, this may be advantageous by reducing selective pressure for resistance and helping maintain the ecological balance of the oral microbiota. However, sustained or repeated application would likely be required to preserve growth inhibition, and the anticariogenic potential may be further enhanced when combined with strategies that provide mechanical plaque removal or complementary bactericidal activity. In this study, a 1% (w/w) concentration of fingerroot extract was used, corresponding to approximately 625 times the MIC and 39 times the MBC values. This concentration was intended to be higher than the MIC and MBC and could promote a clear zone (from disk diffusion or agar well diffusion methods) to evaluate the inhibition of microbial growth for the formulation. Although this concentration facilitated antimicrobial evaluation using agar diffusion methods, it may not directly represent the concentration required under practical clinical conditions, and further studies on dose optimization and safety are necessary. The anticariogenic activity of fingerroot extract against *S. mutans* may involve multiple mechanisms associated with its major flavonoids, particularly PC and PA [9, 10]. Previous studies have suggested that these compounds and fingerroot extract could interfere with biofilm formation, and suppress glucosyltransferase‐related processes involved in extracellular polysaccharide synthesis and dental plaque formation [9, 10, 24–27]. These mechanisms may reduce bacterial adhesion and colonization on tooth surfaces, thereby limiting the cariogenic potential of *S. mutans*. However, the present study did not directly establish a quantitative relationship between flavonoid content and antimicrobial activity, as the observed anticariogenic effect may also involve synergistic contributions from other phytochemicals present in the extract.

### 3.3. Optimal Solvent System for Solubilizing Fingerroot Extract

**FIGURE 4:**
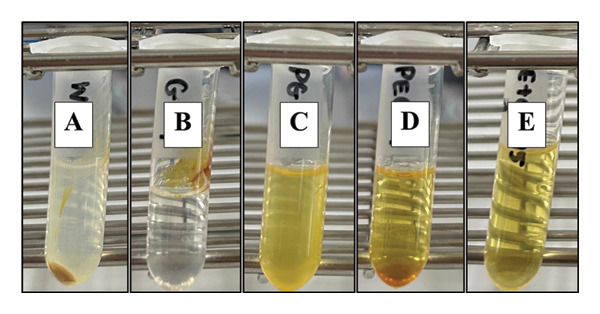
Physical appearance of fingerroot extract in various solvents during recovery determination: (A) water, (B) glycerin, (C) PG, (D) PEG 400, and (E) 95% ethanol.

**FIGURE 5:**
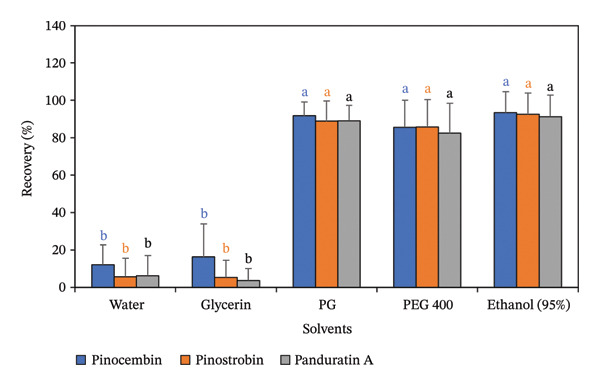
Recoveries of PC, PS, and PA from 1% fingerroot extract prepared using various solvents—water, glycerin, PG, PEG 400, and 95% ethanol—compared to the extraction solvent (85% ethanol). Different letters on the bar graph indicate statistically significant differences (*p* < 0.05) among solvents for the same compound.

The solubilization of herbal extracts is highly influenced by the physicochemical properties of the solvents used. Solvent polarity, viscosity, and hydrogen bonding capacity play crucial roles in determining the extent of solute dissolution. In this study, various solvent compositions were evaluated to understand their effects on the solubilization behavior of key compounds in fingerroot extract. Initially, individual solvents—including water, glycerin, PG, PEG 400, and 95% ethanol—were screened. The percentage recoveries of PC, PS, and PA were calculated relative to 85% ethanol, the optimal extraction solvent for fingerroot. The appearance of 1% fingerroot extract in each solvent is presented in Figure 4. Complete dissolution was observed in 95% ethanol, followed by PG and PEG 400. Although PG resulted in a turbid solution, whereas PEG 400 appeared clear, phase separation was noted. In contrast, the extract was insoluble in water and glycerin. While glycerin, PG, and PEG 400 are all polyols [28], glycerin possesses the highest viscosity, likely hindering mass transfer and consequently limiting solubilization of the extract [29]. The percentage recoveries of PC, PS, and PA in the various solvents are shown in Figure 5. PG, PEG 400, and 95% ethanol demonstrated comparable solubilization capacities, superior to those of water and glycerin. For solvent system optimization, three solvents—water, PEG 400, and 95% ethanol—were selected. Water was chosen for its low cost and favorable safety profile [30], while PEG 400 and 95% ethanol were included due to their effective solubilization of the extract, producing clear solutions. The combination of these three solvents as a cosolvent system might solubilize fingerroot extract.

**FIGURE 6:**
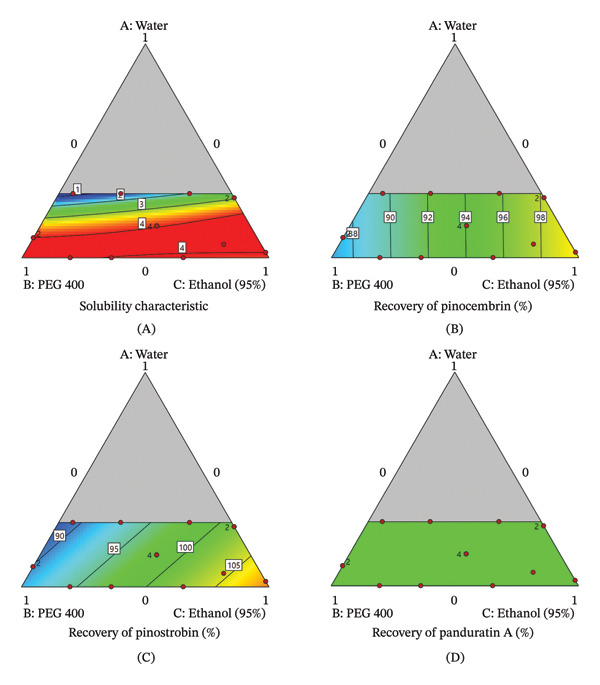
Contour plots of (A) solubility characteristic, (B) recovery of PC, (C) recovery of PS, and (D) recovery of PA, illustrating the effects of solvent mixtures comprising water, PEG 400, and 95% ethanol.

The I‐optimal design has been employed for optimizing solvent systems for herbal extract solubilization [31]. In this study, the I‐optimal design was similarly utilized to optimize the solvent system for fingerroot extract. Factors and responses of the I‐optimal design for the solvent system used to solubilize fingerroot extract are shown in Table 6. The contour plots of solubility characteristics and recoveries of PC, PS, and PA are shown in Figure 6 with the corresponding ANOVA shown in Table 7.

**TABLE 6:** Factors and responses of the I‐optimal design for the solvent system used to solubilize fingerroot extract.

Std	Run	Factors			Responses (*n* = 3)			
		Mass fraction			Solubility characteristic^∗^	PC recovery (%)	PS recovery (%)	PA recovery (%)
		Water	PEG 400	95% ethanol				
2	1	0.094	0.906	0	4	94.77 ± 12.99	94.00 ± 13.19	92.28 ± 12.94
9	2	0.149	0.379	0.472	4	94.88 ± 9.03	94.54 ± 8.46	93.43 ± 8.40
3	3	0.300	0.642	0.0578	1	90.56 ± 2.35	86.18 ± 3.15	85.22 ± 3.36
11	4	0.300	0.171	0.529	2	89.13 ± 2.50	87.49 ± 2.50	86.81 ± 2.51
7	5	0.149	0.379	0.472	4	91.66 ± 3.87	90.82 ± 3.77	89.56 ± 3.76
6	6	0	0.637	0.363	4	83.20 ± 8.46	90.15 ± 9.81	59.49 ± 6.68
12	7	0	0.346	0.654	4	96.49 ± 4.91	104.30 ± 6.53	68.89 ± 4.43
8	8	0.149	0.379	0.472	4	89.56 ± 4.89	95.92 ± 4.41	64.67 ± 1.78
4	9	0	0.804	0.196	4	90.68 ± 0.26	97.25 ± 0.49	62.93 ± 0.30
5	10	0.300	0.449	0.251	1	94.44 ± 8.65	100.28 ± 9.89	67.26 ± 7.05
14	11	0.280	0	0.720	2	100.10 ± 3.62	106.13 ± 4.06	71.60 ± 2.64
1	12	0.094	0.906	0	4	84.80 ± 14.49	91.77 ± 15.36	60.29 ± 11.93
15	13	0.063	0.151	0.786	4	96.00 ± 4.28	102.22 ± 4.70	68.05 ± 3.32
13	14	0.280	0	0.720	4	99.00 ± 6.44	104.57 ± 1.26	71.64 ± 3.42
10	15	0.149	0.379	0.472	4	99.00 ± 1.11	105.44 ± 1.20	70.54 ± 0.92
16	16	0.025	0	0.975	4	104.84 ± 6.53	111.91 ± 6.99	75.25 ± 4.81

^∗^1 to 4 indicate phase separation, sedimentation, turbid solution, and clear solution, respectively.

**TABLE 7:** ANOVA results for the quadratic model of soluble characteristics and the linear models of recoveries of PC and PS.

Source	Sum of squares	df	Mean square	*F*‐value	*p* value
*Solubility characteristic*					
Model	17.5000	5	3.5000	15.5400	0.0002^∗^
Linear mixture	11.9300	2	5.9600	26.4700	0.0001^∗^
AB	3.0700	1	3.0700	13.6200	0.0042^∗^
AC	4.6300	1	4.6300	20.5500	0.0011^∗^
BC	0.0058	1	0.0058	0.0255	0.8762
Residual	2.2500	10	0.2252		
Lack of fit	0.2524	5	0.0505	0.1262	0.9798
Pure error	2.0000	5	0.4000		
Cor total	19.7500	15			

*Recovery of PC*					
Model	220.3500	2	110.1800	5.3500	0.0202^∗^
Linear mixture	220.3500	2	110.1800	5.3500	0.0202^∗^
Residual	267.6800	13	20.5900		
Lack of fit	166.6300	8	20.8300	1.0300	0.5108
Pure error	101.0400	5	20.2100		
Cor total	488.0300	15			

*Recovery of PS*					
Model	424.7600	2	212.3800	6.4300	0.0114^∗^
Linear mixture	424.7600	2	212.3800	6.4300	0.0114^∗^
Residual	429.2800	13	33.0200		
Lack of fit	309.4000	8	38.6700	1.6100	0.3104
Pure error	119.8800	5	23.9800		
Cor total	854.0400	15			

*Note:* An asterisk (^∗^) denotes a significant value.

Results showed that the solubility characteristics followed a quadratic model. An increase in the proportions of water and 95% ethanol initially enhanced the clarity of the solution, which subsequently declined, whereas increasing PEG 400 consistently improved solution clarity. Significant terms influencing solubility included the linear mixture (*p* = 0.0001), the interaction between water and PEG 400 (*p* = 0.0042), and the interaction between water and 95% ethanol (*p* = 0.0011).

The recovery of PC fitted a linear model. Increasing water content had no significant effect on PC recovery, while increasing PEG 400 decreased PC recovery. In contrast, increasing 95% ethanol enhanced PC recovery. The only significant term for PC recovery was the linear mixture (*p* = 0.0202).

The recovery of PS also followed a linear model. Increasing water and PEG 400 resulted in reduced PS recovery, whereas increasing 95% ethanol improved PS recovery. The significant term for PS recovery was the linear mixture (*p* = 0.0114).

Lastly, a contour plot for PA recovery could not be generated, as its values corresponded to the mean recovery across each solvent system, indicating no discernible pattern suitable for model fitting.

**FIGURE 7:**
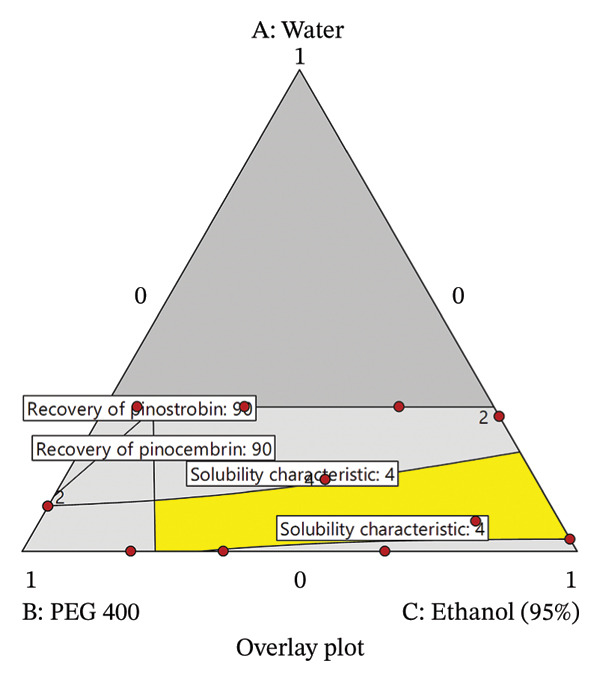
Design space illustrating the region where the solubility characteristic was 4 (clear solution obtained), and at least 90% recoveries of PC and PS were achieved.

The design space in which a clear solution was achieved and the recoveries of PC and PS reached at least 90%, is shown in Figure 7. This design space may facilitate formulation adjustment during product development while maintaining acceptable physicochemical characteristics and flavonoid recovery. This design space provides flexibility for formulation adjustment by identifying solvent compositions capable of maintaining acceptable physicochemical characteristics and flavonoid recovery. In contrast to conventional one‐factor‐at‐a‐time approaches, the I‐optimal design enabled simultaneous evaluation of multiple formulation variables and responses, allowing systematic optimization of extract solubilization, solution clarity, and flavonoid recovery within a single experimental framework. The results demonstrated that the interactions among ethanol, PEG 400, and water significantly influenced both the physical appearance and phytochemical recovery of the formulation. Importantly, the optimized solvent systems allowed reduction of ethanol content while maintaining acceptable extract solubility and flavonoid recovery, which may be advantageous for oral spray formulation development. Verification data demonstrated that all solvent systems and batches produced clear solutions. The percentage errors were relatively low (Table 8), confirming the accuracy and reliability of the predictive model.

**TABLE 8:** Verification data of solvent system for solubilizing fingerroot extract, presented as predicted values, experimental values, and corresponding percentage errors.

Ratio of water:PEG 400:95% ethanol	Responses	Predicted values	Experimental values (*n* = 3)	Error (%)^∗^
12:13:75	Solubility characteristic	Clear solution	Clear solution	—
	PC recovery (%)	97.51	98.54 ± 7.14	1.05
	PS recovery (%)	103.33	97.99 ± 7.16	−5.45
	PA recovery (%)	—	96.25 ± 6.74	—

10:30:60	Solubility characteristic	Clear solution	Clear solution	—
	PC recovery (%)	95.41	100.18 ± 3.98	4.76
	PS recovery (%)	100.83	99.53 ± 3.89	−1.31
	PA recovery (%)	—	98.12 ± 3.89	—

6:60:34	Solubility characteristic	Clear solution	Clear solution	—
	PC recovery (%)	91.72	96.42 ± 1.26	4.87
	PS recovery (%)	96.54	97.47 ± 0.58	0.95
	PA recovery (%)	—	95.96 ± 0.67	—

^∗^Error = (experimental value − predicted value) × 100/experimental value.

### 3.4. Oral Spray Containing Fingerroot Extract

**FIGURE 8:**
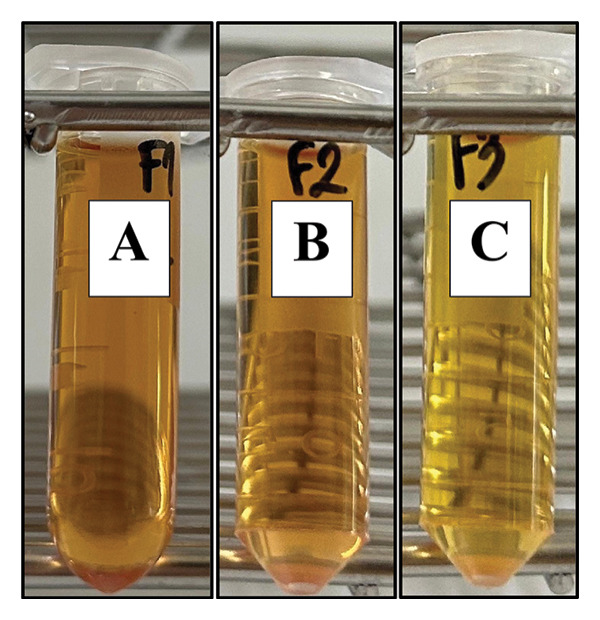
Physical appearance of oral spray formulations containing fingerroot extract in the optimal solvent systems comprising water, PEG 400, and 95% ethanol at the following ratios: (A) 12:13:75 (F1), (B) 10:30:60 (F2), and (C) 6:60:34 (F3).

The oral spray formulations containing fingerroot extract were prepared by additionally incorporating pharmaceutical excipients, such as sweetening agents, flavoring agents, and preservatives. Formulations coded F1 to F3 corresponded to different solvent systems with water:PEG 400:95% ethanol ratios of 12:13:75, 10:30:60, and 6:60:34, respectively. The physical appearance of the oral spray formulations is shown in Figure 8. All formulations appeared clear, with slight color variations.

The oral spray dosage form was selected due to its convenience, ease of administration, portability, and ability to directly deliver active compounds to oral surfaces where *S. mutans* commonly colonizes [2, 32]. Compared with conventional mouthwash formulations, oral sprays may provide more targeted application while requiring a smaller administration volume, which could improve user compliance and convenience [33–35]. Nevertheless, further studies evaluating oral mucosal irritation, cytotoxicity, sensory acceptability, and long‐term safety in human subjects are still required before clinical application.

The potential evaporation of ethanol during spray actuation and subsequent application was considered. Although partial ethanol loss may occur due to its volatility, this is unlikely to substantially affect dose delivery because the antimicrobial efficacy of the formulation is primarily attributed to the fingerroot extract. Furthermore, the spray generates fine droplets that enable rapid deposition of active constituents onto oral surfaces, thereby minimizing the impact of ethanol evaporation on the effective delivery of bioactive compounds.

Physicochemical properties and anticariogenic activity against *S. mutans* of the oral spray formulations are presented in Table 9. The properties varied among formulations. The pH of the formulations ranged from 8.35 to 8.53, which is within the generally accepted range for daily‐use oral care products (7–10, as in toothpaste) and is safe for routine use. This slightly alkaline pH is not expected to damage enamel, dentine, or dental prostheses [36]; however, its long‐term effects on oral microbiota balance and mucosal comfort require further investigation. The combination of reduced ethanol content and controlled pH helps minimize mucosal irritation while maintaining effective anticariogenic activity against *S. mutans*. The viscosity ranged from approximately 10 to 30 cP. The viscosity values were within the recommended limit of not exceeding 100 cP, as higher viscosity can hinder spray performance [37]. Each formulation contained PC, PS, and PA at approximately 0.07%, 0.19%, and 0.07% w/w, respectively. Significant differences in key flavonoid content among formulations may be attributed to the solubilization capacity of the solvent systems; however, all formulations achieved over 90% recovery, as established during the optimization of solvent systems.

**TABLE 9:** Physicochemical properties and anticariogenic activity against *S. mutans* of the oral spray containing fingerroot extract.

Properties	Formulation (*n* = 3)		
	F1	F2	F3
pH	8.53 ± 0.08^a^	8.45 ± 0.05^a,b^	8.35 ± 0.01^b^
Viscosity (cP)	10.00 ± 0.00^c^	14.00 ± 0.00^b^	27.33 ± 1.15^a^
PC content (%)	0.076 ± 0.001^a^	0.071 ± 0.001^c^	0.073 ± 0.001^b^
PS content (%)	0.193 ± 0.002^a^	0.186 ± 0.002^b^	0.194 ± 0.002^a^
PA content (%)	0.073 ± 0.001^a^	0.068 ± 0.001^b^	0.066 ± 0.001^b^
Diameter of clear zone of the formulation (mm)	12.54 ± 1.12^a^	13.72 ± 2.86^a^	11.12 ± 0.62^a^
Diameter of clear zone of the blank formulation (mm)	9.47 ± 0.49^a^	10.21 ± 0.60^a^	Not detected

*Note:* Different superscript letters indicate statistically significant differences (*p* < 0.05) in each formulation’s properties among the formulations.

In Table 9, the blank formulations without fingerroot extract showed clear zones for F1 and F2, indicating that the ethanol content in these formulations contributed some antimicrobial effect. However, the corresponding formulations containing fingerroot extract exhibited larger clear zones, demonstrating that the antimicrobial activity was enhanced by the fingerroot extract itself. Notably, for F3, the blank formulation without fingerroot extract did not produce any clear zone, whereas the F3 formulation with fingerroot extract showed a clear inhibition zone, further confirming the genuine antimicrobial effect of the fingerroot extract. However, the relative contribution of ethanol and fingerroot extract was not quantitatively separated in the present study.

To verify the antimicrobial testing method, the clear zones of positive controls—C20 mouthwash and 0.12% chlorhexidine gluconate solution—were also determined. These exhibited clear zones of 28.67 ± 1.35 mm and 26.88 ± 0.22 mm, respectively.

### 3.5. Stability of Oral Spray Containing Fingerroot Extract

**FIGURE 9:**
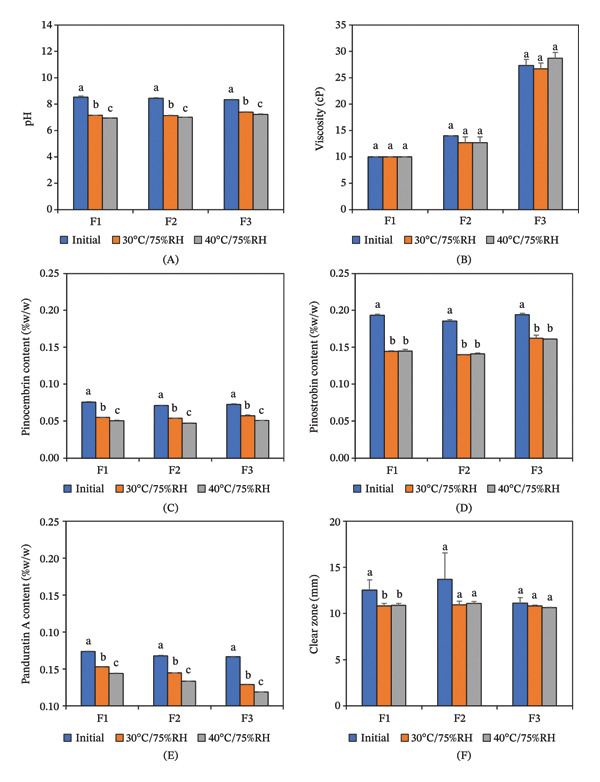
pH, viscosity, PC content, PS content, PA content, and clear zone of oral spray formulations containing fingerroot extract after storage at 30°C/75% RH and 40°C/75% RH for 3 months. Different letters on the bar graphs indicate statistically significant differences (*p* < 0.05) among storage conditions for the same formulation.

Stability data demonstrated that the oral spray formulation remained clear, with no evidence of phase separation or sedimentation. However, the pH of the oral spray formulations significantly decreased to approximately 7.20–7.40 when stored at 30°C/75% RH and to approximately 7.00–7.20 when stored at 40°C/75% RH (Figure 9A). Viscosity remained stable under all storage conditions (Figure 9B). The contents of PC and PA significantly decreased in a temperature‐dependent manner (Figure 9C, E), whereas PS also significantly decreased, but the difference between the 30°C/75% RH and 40°C/75% RH conditions was not statistically significant (Figure 9D). Despite the decline in key flavonoid content, the anticariogenic activity against *S. mutans* remained stable in F2 and F3, although a significant reduction was observed in F1 (Figure 9F). Although a slight decline in each flavonoid content was observed during stability testing, the antimicrobial activity remained stable. This observation may potentially be associated with the contribution of other bioactive constituents in the fingerroot extract or with the remaining flavonoid levels being sufficient to maintain the observed antimicrobial effect. Moreover, the formulation matrix (PEG 400, ethanol, and water) could help preserve the functional activity of the extract despite some quantitative losses. Nevertheless, all formulations retained the ability to inhibit microbial growth. These findings are consistent with a previous study, in which the PC content in fingerroot tablets decreased after stability testing, while antioxidant activity was preserved [38]. The significant decrease in the clear zone against *S. mutans* observed in F1 may be attributed to its high ethanol concentration, which is more susceptible to evaporation from the oral spray formulation compared to the other formulations. Nevertheless, the present stability study was limited to 3 months and did not include degradation kinetic analysis or quantitative measurement of ethanol evaporation, which should be investigated in future studies.

Among the tested formulations, F3—comprising water:PEG 400:95% ethanol at a ratio of 6:60:34—was identified as the best formulation due to its low ethanol content, which is expected to cause less irritation to the oral mucosa [39], and its high potential for further development to enhance formulation stability. Ethanol was included at 34% to ensure solubility and stability of the extract; however, the actual dose delivered per spray is very small, resulting in minimal ethanol exposure per use. This supports the safety of occasional or short‐term use, although continuous daily administration would still require careful consideration.

Although the antimicrobial activity of fingerroot extracts has been previously reported, including studies focusing on extraction optimization and bioactivity characterization, the novelty of the present work lies in its formulation‐oriented approach. This study translates an optimized fingerroot extract into an oral spray dosage form and evaluates its anticariogenic activity against *S. mutans* in the context of formulation composition and delivery. By assessing the antimicrobial performance of the final formulation rather than the extract alone, this work provides application‐relevant insight into the potential use of fingerroot extract as an anticariogenic oral care product.

While the current study demonstrated anticariogenic activity against *S. mutans*, the antimicrobial evaluation was limited to a single bacterial strain and may not fully represent the complexity of multispecies oral biofilms and oral microbiota. Additional studies are necessary to evaluate cytotoxicity on mammalian oral cells (e.g., epithelial cells and fibroblasts), irritation potential, sensory/taste attributes, and quantitative antimicrobial activity to ensure the safety and acceptability of the formulation. Future translational studies should include multispecies biofilm models, CFU reduction assays, biofilm inhibition assays, and in vivo evaluation to better simulate oral conditions and validate efficacy.

## 4. Conclusions

This study successfully demonstrated the integration of UAE and solvent system optimization for the development of an oral spray containing fingerroot extract with anticariogenic activity against *S. mutans*. The extraction process was optimized using a Box–Behnken design, resulting in optimal conditions of 45‐min ultrasonication, a solvent‐to‐solid ratio of 30:1, and 85% v/v ethanol, which yielded the highest levels of bioactive flavonoids—PC, PS, and PA. Subsequently, an I‐optimal design was employed to develop an efficient solvent system composed of water, PEG 400, and 95% ethanol. The solvent systems identified within the constructed design space effectively solubilized the extract and facilitated high flavonoid recovery. These solvent mixtures were used to formulate oral sprays containing appropriate pharmaceutical excipients. Three formulations were selected and demonstrated sustained antimicrobial activity despite reductions in pH and contents of PC, PS, and PA during stability testing. Importantly, these formulations maintained consistent viscosity and inhibition zones against *S. mutans*, indicating preserved functional efficacy. Although additional studies on cytotoxicity, multispecies biofilm models, long‐term stability, and in vivo efficacy are still required, these findings demonstrate the feasibility of integrating extraction optimization, solvent system development, and oral spray formulation within a single formulation strategy. Overall, this study highlights the utility of the design of experiments in optimizing both extraction and formulation processes, contributing to the feasibility of developing a herbal oral spray formulation with potential anticariogenic applications.

## Author Contributions

Chaowalit Monton: conceptualization, methodology, formal analysis, investigation, project administration, software, writing–original draft, writing–review and editing, resource, and supervision. Thaniya Wunnakup: methodology, formal analysis, investigation, and writing–original draft. Jirapornchai Suksaeree: formal analysis, investigation, and writing–original draft. Laksana Charoenchai: formal analysis, investigation, resource, and writing–original draft.

## Funding

No funding was received for this manuscript.

## Conflicts of Interest

The authors declare no conflicts of interest.

## Data Availability

All data generated or analyzed during this study are included in this published article.
